# Telocytes in human oesophagus

**DOI:** 10.1111/jcmm.12149

**Published:** 2013-11-04

**Authors:** Xiaoke Chen, Yonghua Zheng, Catalin G Manole, Xiangdong Wang, Qun Wang

**Affiliations:** a Department of thoracic surgery Zhongshan Hospital, Fudan UniversityShanghai, China; b Department of Respiratory Medicine, Xin Hua Hospital Affiliated to Shanghai Jiao Tong University School of MedicineShanghai, China; c Department of Cellular and Molecular Medicine, ‘Carold Davila’ University of Medicine and PharmacyBucharest, Romania; d Molecular Medicine, National Institute of PathologyBucharest, Romania; e Biomedical Research Center Zhongshan Hospital, Fudan UniversityShanghai, China

**Keywords:** telocytes, telopodes, human oesophagus, VEGF, EGF, stromal synapses, tissue repair

## Abstract

Telocytes (TCs), a new type of interstitial cells, were identified in many different organs and tissues of mammalians and humans. In this study, we show the presence, in human oesophagus, of cells having the typical features of TCs in lamina propria of the mucosa, as well as in muscular layers. We used transmission electron microscopy (TEM), immunohistochemistry (IHC) and primary cell culture. Human oesophageal TCs present a small cell body with 2–3 very long Telopodes (Tps). Tps consist of an alternation of thin segments (*podomers*) and thick segments (*podoms*) and have a labyrinthine spatial arrangement. Tps establish close contacts (‘stromal synapses’) with other neighbouring cells (*e.g*. lymphocytes, macrophages). The ELISA testing of the supernatant of primary culture of TCs indicated that the concentrations of VEGF and EGF increased progressively. In conclusion, our study shows the existence of typical TCs at the level of oesophagus (mucosa, submucosa and muscular layer) and suggests their possible role in tissue repair.

## Introduction

Telocytes (TCs) were recently described as a new type of interstitial cells by Popescu *et al*. 1–18. The existence of TCs was documented in various organs: skin 18,19, brain 21, eye 17, skeletal muscle 7–23, respiratory tract 6–27, heart 3–33, digestive system 11–40, genital tract 16–45 and urinary tract 46,47. Morphologically, TCs have a small cell body with suddenly emerging very long and thin cell prolongations – telopodes (Tps). Tps have particular features, being very thin (less than 0.2 μm) and very long (tens to more than one hundred of μm), of uneven calibre (alternation of thin and dilated segments) with three-dimension spatial arrangement 1–49. Moreover, TCs, by Tps, are physically involved in a complex network, either by homocellular (between TCs), or by heterocellular junctions (junctions between TCs and other cells: blood vessels, muscular cells, nerve cells or connective tissue resident cells) 1–10. On the other hand, previous published data showed the ability for paracrine secretion of TCs for small molecules (NO), and also for shedding vesicles containing peptides (VEGF, IL6) and miRs 9–49. This could be important for short-distance regulations of the surrounding environment. Both physical (long-distance) interaction and chemical (short-distance) interaction are suggestive of a potential role of TCs in intercellular signalling. Recently, a particular attention was given to the tandem TCs – stem cells (SCs) in different organs: lung 6–26, heart 3,10, choroid plexus 21, striated muscle 7 and also skin 18.

The ultrastructure of the oesophageal wall (especially oesophageal stroma) was previously described, with special reference to Interstitial Cells of Cajal (ICCs) 50. As TCs and ICCs are two distinct types of cells, the presence of TCs was omitted at that time 50,51. However, the presence of TCs was recently documented within stroma of different segments of the gastrointestinal tract 14–36. Noteworthy, the oesophageal stroma is an active participant to several disorders 53,54. Moreover, it was previously suggested that TCs, by their ultrastructural features and paracrine secretory potential, might represent the origin of PEComa and GISTs 34 in digestive tract.

Considering previously described close spatial relations of TCs with (new-formed) blood vessels in normal tissue 9, we aimed to assess the presence of TCs in human oesophageal interstitium and also their secretory capacity for angiogenesis associated cytokines. These results could be of great importance for further studies regarding the potential biological functions of TCs.

## Materials and methods

### Patients enrolled in the study

Patient selected for this study fulfilled the following criteria: (*i*) patients who suffered primary oesophagus cancer were confirmed histologically; (*ii *) patients underwent curative surgery, but did not receive any pre-operative treatment; and (*iii *) adjacent oesophagus tissues samples, used as control, were confirmed histologically as the normal. The study was approved by the Ethic Committee of Fudan University, Zhongshan Hospital. All collections of human tissues were performed after obtaining informed consent from the patients.

### Human oesophagus samples

Human oesophagus samples were obtained from patients undergoing thoraco-abdominal incision radical esophagectomy (six patients) or Ivor-Lewis esophagectomy (four patients) for neoplastic oesophagus diseases. Normal human oesophagus samples were collected from the corresponding normal tissues adjacent to resection margins from patients who had no anti-cancer treatment before tumour resection. All samples were examined and confirmed by pathologist. In all, 10 primary oesophagus tissue samples for study were obtained. Human adjacent normal oesophagus tissue samples underwent protocols for (*i* ) histology and immunochemical staining; (*ii* ) electronic microscopy; (*iii* ) fresh tissues for isolation and primary cell culture of TCs.

### Transmission electron microscopy (TEM)

Fresh oesophageal tissue samples were collected in PBS at pH 7.4 and prepared for TEM as previously described 24. Grids were examined in at an acceleration voltage of 80 kV, in JEOL JEM-1230 (Tokyo, Japan) electron microscope. Digital pictures (2048 × 2048 pixels, 4 MB and uncompressed greyscale TIFF files) were obtained by using a high-resolution digital camera Olympus MegaView III connected to the electron microscope.

### Immunohistochemistry (IHC)

Fresh oesophageal tissue samples were fixed with 10% neutral formalin (Shanghai, China) embedded in paraffin. The primary antibodies were used as follows: vimentin, rabbit polyclonal (ab92547; Abcam, Cambridge, MA, USA), 1:100; CD34, rat monoclonal (ab6330; Abcam), 1:100. Negative controls were performed by omitting the primary antibody. Tissue sections were examined and photographed under an Olympus light microscope (BX51) equipped with a digital camera (Olympus dp71; Olympus, Tokyo, Japan). The images were digitally acquired by using the software ImagePro-Express.

### Primary cell culture and vital staining

Oesophageal samples from each patient were prepared for primary cell culture and vital staining as previously described 24. Cells were examined by phase-contrast microscope, under an inverted Olympus phase-contrast microscope (1X51; Olympus) and the images were digitally acquired by using the software CellSens Standard.

### ELISA

The supernatant was collected in 25 cm^2^ plastic culture flasks with primary TCs at 24 and 48 hrs, separately (2 ml of each; *n* = 10) and stored at −20°C. Blank medium hatched in the same atmosphere of cell culturing was collected simultaneously as control (*n* = 10). The concentrations of VEGF and EGF in the supernatant were detected by the method of ELISA with corresponding ELISA kit (eBioscinece, San Diego, CA 92121, USA).

## Results

### Ultrastructure of oesophageal TCs

**Figure 1:**
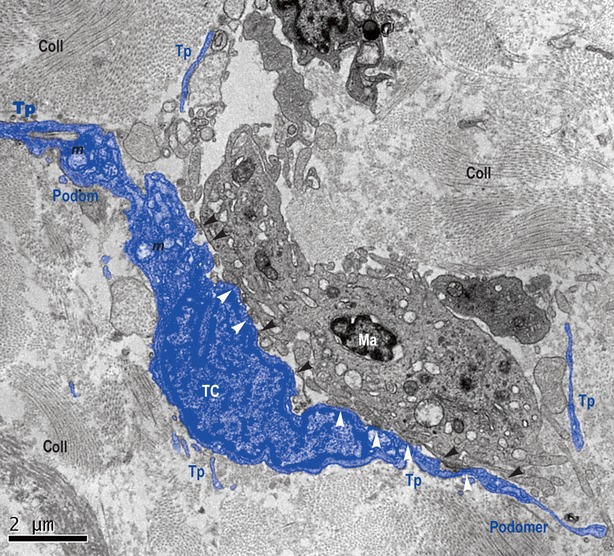
Lamina propria of mucosa of human oesophagus. Transmission electron microscopy. Telocyte (TC) and its Telopodes (Tps) in close spatial relationship with a macrophage (Ma). TC extends its Tps wrapping Ma. The typical silhouette of Tps is obvious: alternation of podoms (dilated segments) and podomers (thin segments). Several direct membrane-to-membrane contacts are visible, either point contacts (black arrow-heads), or planar contacts (white arrow-heads). Coll: collagen; m: mitochondria.

**Figure 2:**
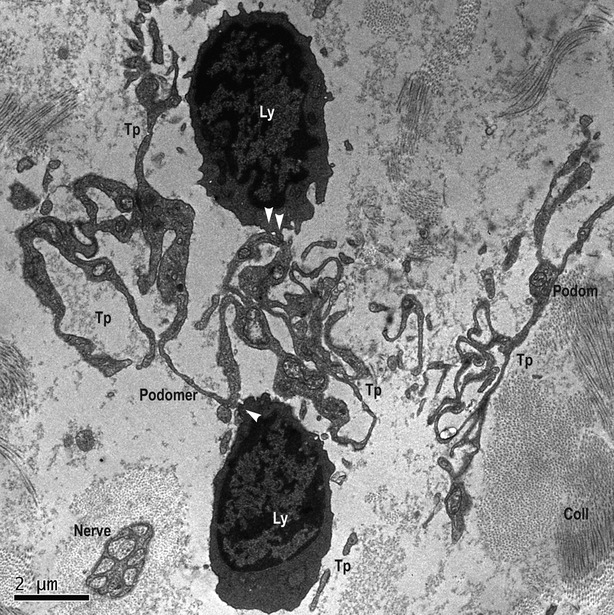
Lamina propria of mucosa of human oesophagus. Transmission electron microscopy. Telopodes (Tp), probably of different Telocytes, having a labyrinthine spatial arrangement. The characteristic alternation of podoms and podomers is obvious. Several point contacts (white arrows) are present between Tps and lymphocytes (Ly), at different levels. Even if not in direct contact with nerve, its close vicinity should not be neglected. coll: collagen.

**Figure 3:**
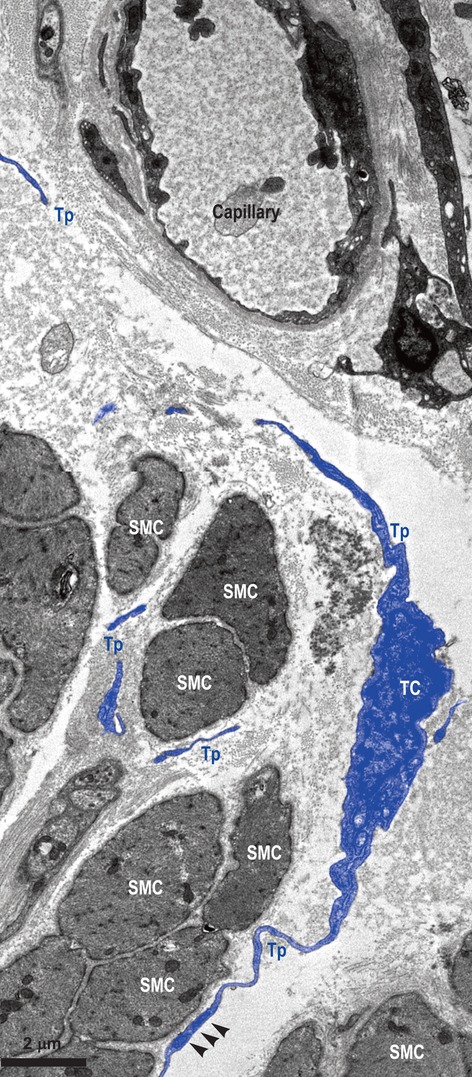
Muscular layer of human oesophagus. Transmission electron microscopy. Telocyte (TC) with two visible Telopodes (Tps) in close vicinity of smooth muscle cells (SMC), and wrapping them. Fragments of (most probably) the same Tp are interposed between SMC and a blood capillary. Small electron-dense nanostructures (arrow-heads) are seen between both membranes – SMC and Tp.

The ultrastructure of the entire thickness of the oesophageal wall was performed. TEM showed the existence of TCs within lamina propria of the human oesophageal mucosa (Figs 1 and 2) as well as in interstitium of the human oesophageal muscular layer (Fig. 3). TCs have a large ovoid nucleus surrounded by a thin rim of cytoplasm (Figs 1 and 3). As it was observed on TEM images, TCs averaged two cellular prolongations – Telopodes (Tps; Figs 1 and 3). Tps are very long and are suddenly emerging from the cell body (Figs 1 and 3). Tps have their key-features: (*i*) very long; (*ii*) beads-on-a-string appearance: alternation of thin segments (*podomers*) – less than 0.2 μm – and dilated segments (*podoms*) 1–49. Podoms are housing the so-called ‘Ca^2+^ uptake/releasing units’ composed of endoplasmic reticulum, mitochondria and caveolae (Fig. 3).

Telocytes and their Tps are in close spatial relationships with nerves (Fig. 2), and establish heterocellular junctions either with immune cells (macrophage – Fig. 1 – and lymphocytes – Fig. 2) or smooth muscle cells (SMC; Fig. 3). In Figure 1, the junctions in between TC and macrophage are either point contacts, or multiple planar contacts. Figure 2 shows many point contacts in between the Tps and neighbouring lymphocytes. Figure 3 shows three small electron-dense nanostructures bridging together the cellular membranes of SMC and Tps’.

Since Tps are eminently three-dimension structures with a 3D spatial orientation, it was improbable their full-length to be encompassing in the same extremely thin plane of section (basically bi-dimensional), as for TEM. Thus, Figures 3 present interruptions in the Tps continuum. However, Figure 2 shows the labyrinthine pattern of Tps arrangement, and, on the other hand, shows a characteristic location of TCs and Tps: in close vicinity of a capillary, between capillary and its specific target (here, SMC).

### Immunohistochemistry

**Figure 4:**
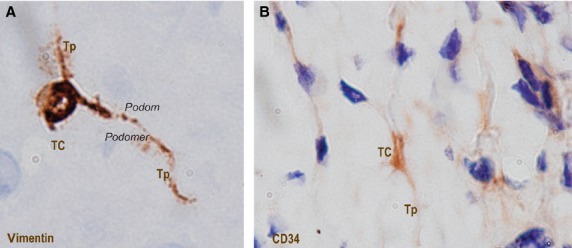
Submucosa of human oesophagus. Immunohistochemistry. Vimentin-positive cells (A) and CD34-positive cells (B). Both cells morphologies are very evocative for Telocytes (TC): small cell body (A and B) with long cell processes (A and B) – Telopodes (Tp), with their characteristic moniliform silhouette (alternation of podoms and podomers) are clearly visible (A). Original magnification: 1000×.

Under light microscopy, vimentin-positive cells were seen within human oesophageal submucosa (Fig. 4A). Positive cells have several suggestive mark-ups for TCs: small cell body and few very long, and very thin moniliform cell prolongations – Tps – with characteristic alternations of podoms and podomers. The positive expression for vimentin is present on cell body and Tps also. The positivity for CD34 was also tested in oesophageal submucosa (Fig. 4B). IHC for CD34 shows positive expression on cells of similar morphology with vimentin-positive TCs. TCs were previously reported positive for CD34. CD34-positive human oesophageal TCs have a small cell body with very long slender cellular prolongations.

### Primary cell culture and vital staining

**Figure 5:**
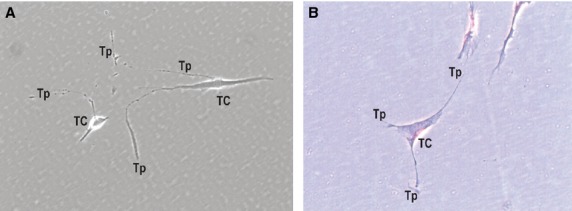
Primary culture of human oesophagus Telocytes. Phase-contrast microscopy. Vimentin-positive cells (A) and CD34-positive cells (B). Both cell morphologies are very evocative for Telocytes (TC): small cell body (A and B) with long cell processes (A and B) – Telopodes (Tp), with their characteristic moniliform silhouette (alternation of podoms and podomers), are visible (A). Original magnification: 1000×.

Telocytes were successfully maintained in primary culture and could easily be identified before reaching confluence. Starting with the 3rd day in culture, TCs appeared with characteristic long, moniliform processes (Tps; Fig. 5A). Observed under phase-contrast microscopy, in cell cultures, TCs have a variable number of Tps (2–3); thus, the shape of the cell body varies (from spindle to triangular). We used Giemsa vital staining on primary cell cultures of TCs (Fig. 5B). In cell culture stained with Giemsa, living TCs have very long Tps of uneven calibre (alternation of podoms and podomers).

### Detection of cytokines in the supernatant

**Figure 6:**
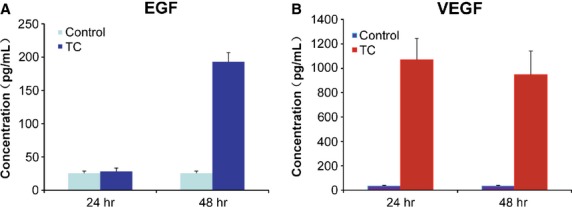
The concentrations of EGF and VEGF detected by ELISA. (A) The concentration of EGF in the supernatant of cultured telocytes was slightly changed at 24 hrs, but abruptly increased (almost four times) at 48 hrs, in comparison with control. (B) The concentration of VEGF in the supernatant of cultured telocytes was significantly higher than in control at 24 hrs, and slightly decreased at 48 hrs.

The supernatant of the primary cultures of TCs was collected and we measured the concentrations of VEGF and EGF. We used a double-antibody sandwich ELISA. The results are shown in Fig. 6A and B. The concentration of EGF had little change at 24 hrs (28 ± 5 pg/ml), being compared with control (DMEM without FBS); however, it increased several folds at 48 hrs (193 ± 14 pg/ml) than control (26 ± 3 pg/ml; Fig. 6A). The concentration of VEGF was significantly higher (1069 ± 175 pg/ml) than control (DMEM without FBS) (34 ± 6 pg/ml) at the first 24 hrs, and decreased slightly at 48 hrs (948 ± 194 pg/ml; Fig. 6B).

## Discussion

Telocytes are, by themselves, a distinct type of interstitial cells whose presence was documented by Popescu's group within interstitium of various organs 1–18. We report here the presence of TCs in lamina propria of human oesophageal mucosa, submucosa, as well as in muscular layer. Previous studies have shown, by immunofluorescence and immunohistochemistry, the existence of TCs positive for PDGFRα and CD34 within submucosa and in the interstitium of muscular coat of the oesophagus 40. Ultrastructural studies, published hitherto, regarding oesophageal interstitium, neglected the presence of TCs (this distinct new type of interstitial cells), being focused mostly on the presence of ICC 51–52. Within interstitium of lamina propria of submucosa, TCs appear in close relationships with immune cells (macrophages and lymphocytes). Moreover, TCs seem to be involved in stromal networks by interconnecting such cells through heterocellular junctions (‘stromal synapses’ 56). These heterocellular junctions are either point contacts (Figs 1 and 2), or planar contacts (Fig. 2). At the level of lamina propria of oesophageal mucosa, TCs could integrate signals, by their junctions to immune cells. In this respect, we can expect oesophageal TCs (or TCs network) to behave as an immune system modulator, interrelating immune cells in interstitium context and providing functional support at the level of oesophagus.

On the other hand, within the muscular coat of the oesophagus, TCs seem to have strategic positions, enwrapping bundles of SMC, or being situated between SMC and blood vessels. We can state that these images somehow recapitulate the particular spatial distribution of TCs found in other organs 1,5.

Immunohistochemistry labelling for vimentin and CD34 demonstrates that in the submucosa of oesophagus resides a population of vimentin/CD34-positive cells having evocative morphology for TCs. The positive expression for these markers is present at the level of cell body and also their prolongations, and can discriminate TCs from other interstitial cells (fibroblasts, mesenchymal stem cells, *etc*.). Also, cell culture obtained from submucosa of human oesophagus shows a major population of interstitial cells with morphology typical of TCs, as it was stained for Giemsa (but also methylene blue and Janus Green B – data not included).

In this study, by ELISA testing, we showed that, *in vitro*, TCs produce measurable amounts of VEGF and EGF. Using mass spectrometry (SELDI-TOF-MS), previous published data showed that the detected levels of IL-6 and VEGF in TCs culture increased with passage number (to 964.47 pg/ml for IL-6 and 262.20 pg/ml for VEGF) 49. In this study, by ELISA, we show that in primary culture of TCs, after 48 hrs, the concentrations of VEGF and EGF were high (193 ± 14 pg/ml for EGF and 948 ± 194 pg/ml for VEGF respectively). This confirms (and complete, also) previous published data regarding the secretory profile of TCs. The secretory levels of TCs for these cytokines, correlated with our TEM data on TCs close spatial relations with blood vessels and SMC in human oesophagus might suggest TCs involvement in neoangiogenesis or cell differentiation.

In conclusion, our study clearly shows the existence of TCs within interstitium of mucosa, submucosa and muscular layer of oesophagus and suggests possible role(s) of TCs in pathogenesis of several oesophageal disorders that involve (neo)angiogenesis and/or cell differentiation.
